# Image Analysis of Endosocopic Ultrasonography in Submucosal Tumor Using Fuzzy Inference

**DOI:** 10.1155/2013/329046

**Published:** 2013-08-19

**Authors:** Kwang Baek Kim, Gwang Ha Kim

**Affiliations:** ^1^Department of Computer Engineering, Silla University, Busan 617-736, Republic of Korea; ^2^Department of Internal Medicine, Pusan National University School of Medicine and Biomedical Research Institute, Pusan National University Hospital, Busan 602-739, Republic of Korea

## Abstract

Endoscopists usually make a diagnosis in the submucosal tumor depending on the subjective evaluation about general images obtained by endoscopic ultrasonography. In this paper, we propose a method to extract areas of gastrointestinal stromal tumor (GIST) and lipoma automatically from the ultrasonic image to assist those specialists. We also propose an algorithm to differentiate GIST from non-GIST by fuzzy inference from such images after applying ROC curve with mean and standard deviation of brightness information. In experiments using real images that medical specialists use, we verify that our method is sufficiently helpful for such specialists for efficient classification of submucosal tumors.

## 1. Introduction

Recently, many digestive diseases are found in the early stage due to increasing usage of upper gastrointestinal endoscopy. One of those disease groups is a submucosal tumor (SMT). An SMT is a spherical or hemispheric lesion projected toward the lumen of which main lesion exists below the mucosa and its surface is covered with normal gastrointestinal mucosa. Among those SMTs, leiomyoma, cyst, fibroma, lipoma, and hemangioma are benign tumors, but gastrointestinal stromal tumor (GIST), leiomyosarcoma, and lymphoma have malignant potential. 

Because native-eye findings of the endoscopic image are very similar and histological confirmation is mainly not possible by endoscopic biopsy only, medical specialists have great difficulty in classifying them correctly. Endoscopic ultrasonography (EUS) overcomes such difficulty in diagnosing SMTs, and it is also used for staging malignant tumors in the digestive tract [1, 2].

Most SMTs are benign. However, benign SMTs are not easily distinguished from malignant SMTs, and even if they are truly benign, there is no agreement among specialists in how frequently a followup is needed or when operative treatment should be given to the patient. 

GISTs have a risk of metastatic relapse, especially in the peritoneum and liver, after surgery for localized diseases [3, 4]. Therefore, every GIST is now considered as potentially malignant, and so all GISTs may need to be resected, even small intramural lesions of the stomach [5]. 

In practice, the differentiation of GISTs from benign SMTs is essential to clinical management. However, the studies for distinguishing between GISTs and other benign mesenchymal tumors by EUS are still only a few [6, 7]. 

In addition, there are limitations in the analysis of the characteristic EUS features because of poor interobserver agreement by subjective interpretation of EUS images [8, 9]. Therefore, if an objective analysis for EUS images would be possible especially by means of computer-assisted image analysis, the previous limitation might be overcome.

Thus, in this paper, we propose a method to extract areas of GIST and lipoma automatically from the standardized ultrasonic image to assist those endoscopists. We also propose an algorithm to differentiate GIST from non-GIST by fuzzy inference [3] from such images after applying an ROC curve with mean and standard deviation of the brightness information.

## 2. Materials and Methods

### 2.1. Extracting Gastrointestinal Stromal Tumor (GIST)

EUS was performed using a radial scanning ultrasound endoscope (GF-UM2000; Olympus, Tokyo, Japan) at 7.5 MHz. All the examinations were performed under intravenous conscious sedation (midazolam with or without meperidine). Scanning of the tumor was performed after filling the stomach with 400–600 mL of deaerated water. At least 10 endosonograms were recorded for each lesion, and these images were digitally saved in the Windows bitmap format.

Reviewing the EUS images was performed by a single experienced endosonographer (Kim et al. [6]) who was kept “blinded” to the final diagnosis, and only one highest quality EUS image for each lesion was selected for further analysis, which was performed on a standard desktop computer.

GIST is a mesenchymal tumor with malignant potential found in the stomach, and small and large intestine. The majority (60~70%) is found in the stomach. Therefore, we included gastric GISTs located in this study. 

**Figure 1:**
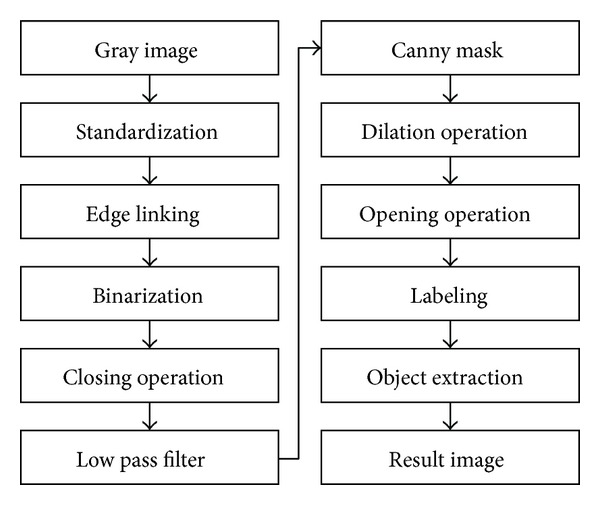
Process for extracting GIST.

Figure 1 shows the overall process for extracting GIST by the proposed method.

There are too many edges in the GIST area from the standardized EUS image [10–12], but the boundary lines could be removed according to the characteristic that boundary lines have too high or too low brightness.

For pixels that have sufficient brightness (experimental threshold above 30), we apply an edge linking method that connects the current pixel to adjacent pixels if formula (1) is satisfied. The experimental threshold Th in our study was 130:

(1) $$\begin{array}{c} \left|\Delta G\left(x,y\right)-\Delta \left(G^{\prime }\left(x^{\prime },y^{\prime }\right)\right)\right|\leq \text{Th}. \end{array}$$

**Figure 2:**
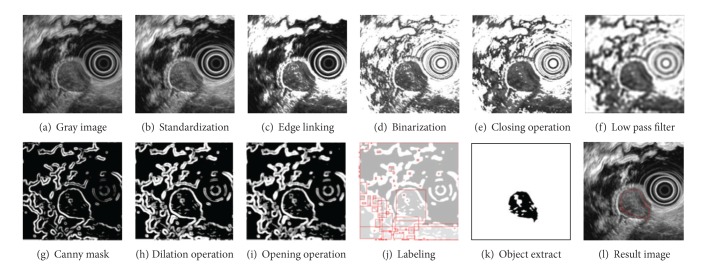
GIST extraction.

Then we remove low brightness pixels in the GIST area by setting those pixels' brightness as 255 if the brightness is no higher than 40.  Figure 2(d) shows the result of the proposed procedures.

Also, noise removal is followed by applying a morphological closure operation in order to fill the gap or little holes while maintaining the size and the shape of the object as shown in Figure 2(e). The resulting image is smoothed by a Butterworth low-frequency filter for irregular edges in the tumor area as shown in Figure 2(f). Boundary lines are extracted by using a noise-insensitive Canny mask to remove minute noise. From that noise-free Figure 2(g), we apply a dilation operation to reconnect unexpectedly disconnected boundary lines during the preceding process and also an opening operation to maintain the original size of objects after such noise removal as shown in Figure 2(i). 

Then we apply a GrassFire algorithm to label them as shown in Figure 2(j) as all pixels in the same object have the same identification number and remove objects including lens and other subtle noise. Finally, the GIST area is extracted by taking objects that have high density of pixels as demonstrated in Figure 2(l) to finish the process.

### 2.2. Extracting Lipoma

Lipoma is a well-capsulated benign tumor consisting of matured adipocyte. It can be found everywhere but it is usually seen in the subcutis of normal adipose tissue such as thigh, arm, and torso. Lipoma is one of the most frequent benign tumors found in the soft tissue among age 40~60, and it is often found in the stomach during endoscopy.

Lipoma area usually has high brightness, and its boundaries are clear. We apply histogram smoothing to regulate brightness distribution of lipoma area. We control lower brightness of pixels (<75) as giving brightness zero in order to remove dark noise. 75 is an experimental threshold that is the lowest brightness of lipoma area.

**Figure 3:**
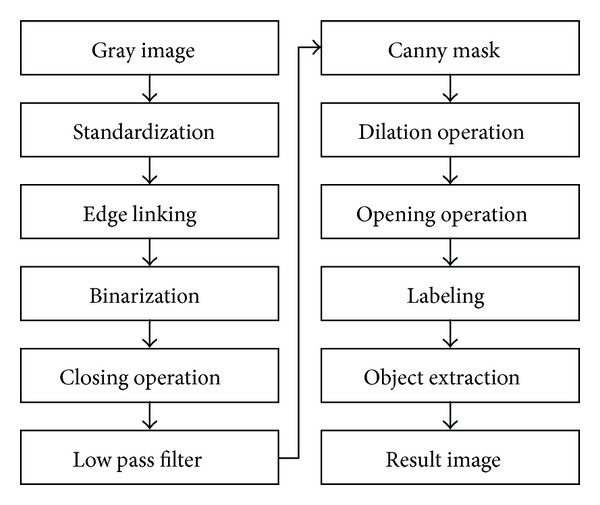
Process for extracting lipoma.

**Figure 4:**
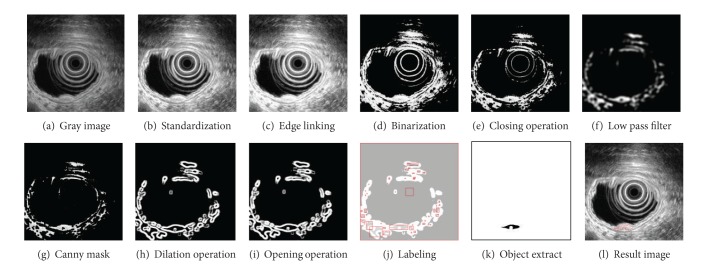
Lipoma extraction.

The process of noise removal and object extraction is similar to that of the GIST case explained in Section 2.1. Figures 3 and 4 demonstrate the diagram and corresponding treated images.

### 2.3. Classifying Tumor by Fuzzy Inference

In our method, an endoscopist chooses the tumor area on the standardized EUS image. Then we apply the average brightness (MEAN) and standard deviation (SD) information to the ROC curve [13, 14] which visualizes the prediction rate of true positivity and false positivity. The results are used as the membership function intervals of our fuzzy theory [15, 16]. By applying the ROC curve to the MEAN and SD of the chosen tumor area, we obtain Table 1, and those results are used to establish fuzzy membership function intervals.

**Figure 5:**
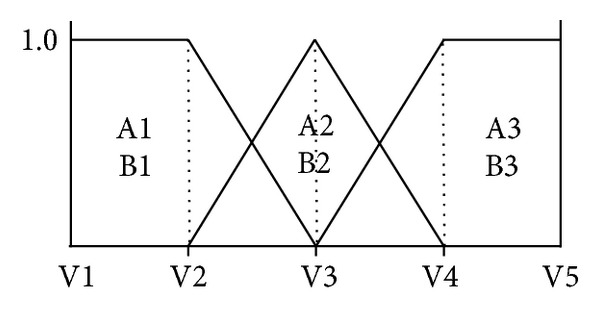
First half membership functions.

Figure 5 denotes the membership functions where A denotes MEAN and B denotes SD.

In Figure 5, intervals of V1, V2,…, V5 are categorized as reported in Table 2.

After computing the degree of membership using Figure 5, we use fuzzy inference rules as follows.

 IF A1 and B1 then G1
 IF A1 and B2 then G1
 IF A1 and B3 then G1
 IF A2 and B1 then G1
 IF A2 and B2 then G2
 IF A2 and B3 then G2
 IF A3 and B1 then G2
 IF A3 and B2 then G2
 IF A3 and B3 then G3

**Figure 6:**
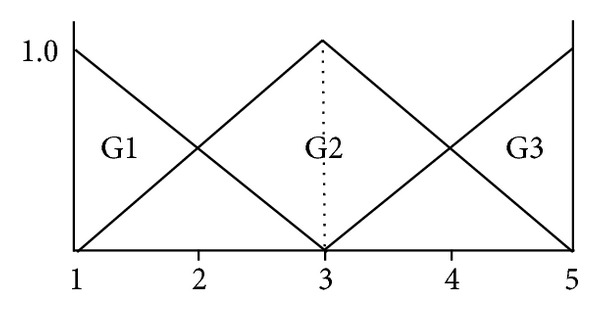
Membership functions for tumor classification.

We use popular Min_Max reasoning [17] and apply it to the membership degree of GIST as shown in Figure 6 and use centroid method as a defuzzifier as shown in formula (2). Finally, the class of tumor is defined by criteria as shown in Table 3:

(2) $$\begin{array}{c} W_{z}=\frac{\sum \mu (X_{i})X_{i}}{\sum \mu (X_{i})}. \end{array}$$

## 3. Results

In experiments, we used real EUS images from endoscopists for three different types of tumor, ten cases per type. The software is written in VC++ 2005 on notebook with Intel Pentium dual-core 2 GHz CPU and 3 GB RAM.

**Figure 7:**
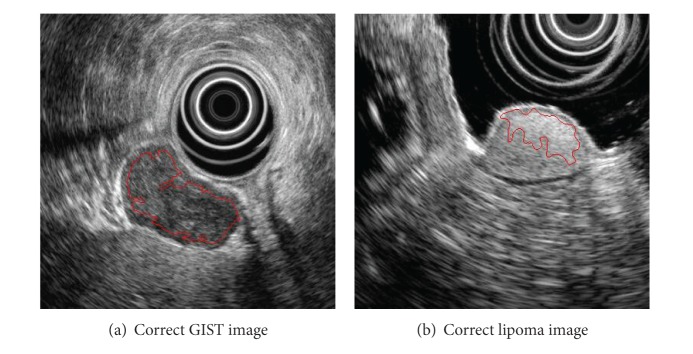
Correct tumor extraction.

The result of GIST and lipoma extraction is shown in Table 4. And Figure 7 demonstrates successful extraction cases of GIST and Lipoma.

**Figure 8:**
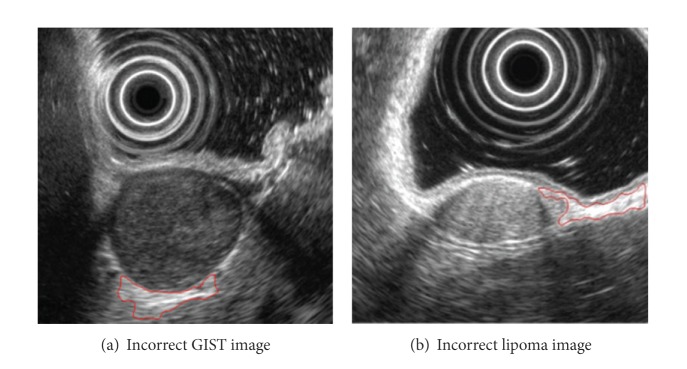
Incorrect tumor extraction.

Two failed cases for GIST are due to unexpectedly high density of pixels of noises, and one failed case in extracting lipoma is due to including unnecessary objects when we extract edges with the Canny mask. Such cases are shown in Figure 8.

Table 5 shows the classification results of three different tumors by fuzzy inference. We take this as two-class problem in that we are only interested in GIST and non-GIST. There exist one or two failed cases for each class but overall, it is sufficiently accurate for endoscopists as an auxiliary tool.

## 4. Conclusions

In this paper, we propose a method to extract GIST and lipoma from EUS images and a classification scheme with fuzzy inference whether it is a GIST or not. From the standardized EUS images, we apply various image processing algorithms such as binarization, morphological operations, GrassFire algorithm, Canny mask, smoothing, and so forth. in order to remove noise. Then a target tumor is extracted by the characteristic of high density of pixels. We also propose a method to discriminate GIST from non-GIST tumor with fuzzy inference rules.

In experiments which used real clinical data, the extraction of GIST and lipoma is not yet fully successful; the accuracy is about 85%. However, the classification of tumors is almost correct overall, where 27 of 30 cases have been correctly classified. This experience stimulates us to develop more accurate extraction algorithm in the future.

## Figures and Tables

**Table 1:** Results for the ROC curve.

	Sensitivity	1-specificity	AUC
Mean	65.12	0.896	0.091
SD	74.97	0.917	0.273

**Table 2:** Membership function intervals.

	V1	V2	V3	V4	V5
Mean					
A1	0	55	65		
A2		55	65	75	
A3			65	75	255

SD					
B1	0	65	75		
B2		65	75	85	
B3			75	85	255

**Table 3:** Criteria for tumor classification.

1 ≤ *W*_*z*_ ≤ 2	Non-GIST (cyst)
2 ≤ *W*_*z*_ ≤ 4	GIST
4 ≤ *W*_*z*_ ≤ 5	Non-GIST (lipoma)

**Table 4:** Tumor extraction results.

	Successful/total
GIST	8/10
Lipoma	9/10

**Table 5:** Results of fuzzy analysis.

	Successful/total
GIST	8/10
Non-GIST	19/20
